# Washington R. Handy

**Published:** 1858-04

**Authors:** 


Obituary.—It is with sincere sorrow we are called upon to announce the death of our much lamented friend and colleague, Professor Washington R. Handy, M. D., with whom the senior editor had been associated in the Baltimore College for about seventeen years. He died on Monday, the 4th of January, 1858, after a very severe and painful illness of about three weeks, but his health for several years previous had been feeble. Dr. H. was a man of retiring manners, a sincere friend, strictly honorable in all his dealings with his fellow man, possessed of unbending integrity, and conscientiously faithful in the discharge of every duty which he undertook. In other words he lived and died a Christian. But for a fuller portraiture of his character, the reader is referred to the Valedictory in another part of the present number of the Journal, delivered to the graduating class at the last commencement of the Baltimore College of Dental Surgery, by Professor Piggot. Sen. Ed.

Immediately after the decease of Professor Handy, the Faculty of the College met and passed the following resolutions:

At a meeting of the Faculty of the Baltimore College of Dental Surgery, convened on occasion of the death of Professer W. R. Handy, the following resolutions were adopted:

*Resolved,* That this faculty have learned the death of their colleague, Dr. Handy, with sincere sorrow.

*Resolved,* That, whilst we bow with the profound submission which becomes us to the sovereign will of Almighty God, we deeply deplore the loss of a most valuable colleague and highly esteemed personal friend.

*Resolved,* That we tender our sincere condolence to the relations of the deceased, in their severe affliction

Signed, by order, P. H. Austen,
Dean of the Faculty.

The students also called a meeting and passed the following resolutions:

The Students of the Baltimore College of Dental Surgery, having been informed in a most touching manner by Professor Austen of the death of Professor Washington R. Handy, immediately organized a meeting to express in a suitable manner their sense of this great affliction, and to offer to the bereaved friends of the deceased their sympathy and condolence. The following preamble and resolutions were adopted:

*Whereas,* In the short time we have had the privilege of knowing Dr. Handy, we have not only conceived the highest esteem for him as a man of science, and a kind and efficient instructor, but have also seen in him such evidence of unbending integrity and unvarying kindness of heart as prove to us how dear he must be to those who have been blessed with his intimate acquaintance, and

*Whereas,* It has pleased Providence to take him from our midst, in a manner so afflicting and terrible, that his final dissolution seemed a merciful relief from the horrors which preceded it, and

*Whereas,* While we feel most weighty cause of sorrow at our own loss in this great affliction, we cannot but be sensible how much greater must be the grief of his near and loved friends; it is therefore

*Resolved,* That while it becomes us and all the wide circle whom this affliction bereaves to bow to the will of God, yet we cannot fail to recognize in this dispensation one of the heaviest sorrows that could have embittered the remembrance of our collegiate days.

*Resolved,* That we shall always cherish the liveliest sense of gratitude for the instructions we have been permitted to receive from Dr. Handy, and the warmest recollection of his many virtues and graces, as christian, gentleman and scholar.

*Resolved,* That as a class we respectfully offer to the immediate friends and relatives of Dr. Handy our warmest sympathy in this heavy affliction; while we humbly trust they find in the memory of their loved one's virtues, and the assurance of his infinite gain, higher and more perfect consolation.

*Resolved,* That four members of the class be appointed to accompany the remains of Dr. Handy to Newark, Delaware, where we understand they will be interred.

*Resolved,* That in token of respect for the deceased, the class wear a badge of mourning for thirty days.

*Resolved,* That a copy of these resolutions be transmitted to the relatives of the deceased, and that the same be published. Signed by the committee of the class.

J. G. Russell, M. D. C. S. Hurlbert,

C. W. Cadden, M. D. J. Smith Dodge, Jr., M. D.

Thomas O. Hills,

